# C-Reactive protein reactions to glucose-insulin-potassium infusion and relations to infarct size in patients with acute coronary syndromes

**DOI:** 10.1186/s12872-015-0153-7

**Published:** 2015-12-03

**Authors:** Hadeel Alkofide, Gordon S. Huggins, Joni R. Beshansky, Robin Ruthazer, Inga Peter, Madhab Ray, Jayanta T. Mukherjee, Harry P. Selker

**Affiliations:** Clinical and Translational Science Graduate Program, Sackler School of Biomedical Sciences, Tufts Clinical and Translational Science Institute, Tufts University, Boston, MA USA; Department of Clinical Pharmacy, College of Pharmacy, King Saud University, Riyadh, Saudi Arabia; MCRI Center for Translational Genomics, Molecular Cardiology Research Institute, Tufts Medical Center, Boston, MA USA; Center for Cardiovascular Health Services Research, Institute for Clinical Research and Health Policy Studies, Tufts Medical Center, Boston, MA USA; Regulatory and Clinical Research Management, Department of Health Sciences, Regis College, Weston, MA USA; Tufts Clinical and Translational Science Institute, Tufts University, Boston, MA USA; Department of Genetics and Genomic Sciences, Icahn School of Medicine at Mount Sinai, New York, NY USA

**Keywords:** Acute coronary syndromes, Glucose-insulin-potassium (GIK), Inflammation, C-reactive protein, Metabolic therapy

## Abstract

**Background:**

Some benefits of glucose-insulin-potassium (GIK) in patients with acute coronary syndromes (ACS) may be from an anti-inflammatory effect. The primary aim of this study was to assess the impact of GIK administration early in the course of ACS on inflammatory marker C-reactive protein (CRP) levels. A secondary aim was to investigate the association between CRP and 30-day infarct size.

**Methods and Results:**

Retrospective analysis of participants with ACS randomly assigned to GIK or placebo for at least 8 h in the IMMEDIATE Trial biological mechanism cohort (*n* = 143). High sensitivity CRP (hs-CRP) was measured at emergency department presentation, and 6 and 12 h into infusion. Logarithmically transformed hs-CRP values at 12-hours were lower with GIK vs. placebo (mean =0.65 mg/L in GIK, 0.84 mg/L in placebo), with a marginal trend toward significance (*P* = 0.053). Furthermore, using mixed models of hs-CRP, time, and study group, there was a significant increase in hs-CRP levels over time, but the rate of change did not differ between treatment arms (*P* = 0.3). Multivariable analysis showed that an elevation in hs-CRP, measured at 12 h, was an independent predictor of 30-day infarct size (β coefficient, 6.80; *P* = 0.04) using sestamibi SPECT imaging.

**Conclusions:**

The results of this study show no significant effect of GIK on hs-CRP. In addition our results show that in patients with ACS, hs-CRP measured as early as 12 h can predict 30-day infarct size.

**Electronic supplementary material:**

The online version of this article (doi:10.1186/s12872-015-0153-7) contains supplementary material, which is available to authorized users.

## Backgrounds

Glucose -insulin-potassium (GIK) infusion as metabolic therapy can reduce damage to myocardial cells in the setting of ischemia or infarction [1–4]. Since its introduction in the early 1960s [5], GIK treatment has been assessed in both animal models and human studies. However, the role of GIK in patients with acute myocardial infarction (MI) remains controversial. While several clinical trials have shown no benefit of GIK treatment in patients with acute MI [6–10], other studies have reported benefit [11–15]. Most recently, the IMMEDIATE (Immediate Myocardial Metabolic Enhancement During Initial Assessment and Treatment in Emergency care) Trial failed to show significant differences in the outcome of progression to MI among patients presenting with acute coronary syndromes (ACS); but it did show that GIK was associated with lower rates of the composite outcome of cardiac arrest and/or in-hospital mortality, and with smaller infarct size [15]. The IMMEDIATE Trial is distinguished from other trials by the early administration of GIK prior to arrival at the hospital [15]. In previous studies, GIK administration was delayed until hospital admission [6–10], with a median time of 6 h from symptom onset to GIK initiation in one study [9].

While the mechanisms underlying GIK’s effects in patients with ACS are unknown, several pathways have been suggested, including a metabolic pathway by promoting of glycolysis, and reducing circulating free fatty acid (FFA) levels, and through anti-inflammatory effects [16–18]. Inflammation contributes to myocardial damage in ischemia, infarction, and reperfusion [19]. The acute phase reactant C-reactive protein (CRP) is released in response to inflammation in chronic coronary artery disease and in acute MI [19]. High CRP levels after acute MI predict infarct expansion and plaque rupture [20–22]. Whereas a reduction in the rise of CRP levels has been shown to indicate the efficacy of thrombolytic therapy and a patent infarct-related coronary artery [23, 24]. Continued elevations in CRP portend increased risk of mortality, even in the presence of currently available therapies for ACS [25].

In the setting of ACS, GIK has been demonstrated to reduce serum markers of inflammation, including CRP in one study [18] but not in others [26, 27]. Here, in the largest study to date of very early administration of GIK for ACS, the IMMEDIATE Trial, we tested the hypothesis that GIK was associated with smaller CRP elevations compared to those treated with placebo. Related to this, we further hypothesized that the changes in CRP levels following presentation with an ACS correlate with 30-day infarct size.

## Methods

### Study sample

This study analyzed data collected from participants enrolled in the IMMEDIATE Trial, the methodology of which has been published elsewhere [15]. In brief, it was a randomized, placebo-controlled, double-blind clinical effectiveness trial of GIK, from December 2006 through July 2011, in which paramedics, aided by electrocardiograph (ECG)-based decision support, randomized and enrolled 871 participates aged ≥30 years with high probability of ACS [15]. Participants were given either the GIK solution (30 % glucose, 50 U/L of regular insulin, and 80 mEq of KCl/L) intravenously at 1.5 mL/kg/h for 12 h, or an identical-appearing placebo as 5 % glucose solution. In the study, the median time from symptom onset to initiation of infusion was 90 min [15]. This investigation was based on the IMMEDIATE Trial biological mechanism cohort (“biocohort”), which comprised of participants who consented to participate in this biocohort, confirmed as having ACS, and treated for at least 8 h with study drug. Confirmed ACS (acute MI or angina pectoris) was determined by site investigator review and then independently adjudicated by the study clinical events committee who were blinded to study group, glucose, and potassium results. Enrolment in the biocohort began after the trial was started and only included 6 out of 13 study centers. The study protocol and analyses were approved by the Tufts Institutional Review Board (IRB) and by the IRBs responsible for each of the IMMEDIATAE Trial study sites.

### Data collection

During the 12-hour infusion of study drug, glucose, insulin, FFA, and high sensitivity CRP (hs-CRP) levels were tested at three times: 1) the initial measurement, which was drawn as soon as feasible after hospital arrival; 2) 6 h after start of study drug; and 3) 12 h after start of study drug, or upon discontinuation of the infusion. Hemoglobin A1C was measured on admission and at 30 days, and infarct size was measured at 30 days by sestamibi SPECT imaging. The Trial’s core laboratory interpreted the nuclear studies, and the core laboratory of Tufts Clinical and Translational Science Institute performed the hs-CRP measurements. Other covariates measured in the biocohort include demographic data, vital signs, medical history, and medications used at home, in the hospital, or upon discharge.

### Data analysis

Statistical analyses were performed using R, version 2.15.2. Descriptive statistics were used to describe baseline characteristics. All tests were two-sided, using alpha <0.05 to determine statistical significance. Serum hs-CRP levels ranged widely and their distribution was highly skewed to lower levels; therefore hs-CRP concentrations were logarithmically (base 10) transformed in all further analyses. Linear regression models were used to assess the relationship between initial hs-CRP measurements and baseline characteristics; variables significantly associated with initial hs-CRP levels were adjusted-for in the analysis. The independent sample t-test was used to detect unadjusted differences in cross-sectional hs-CRP levels between GIK and placebo. In addition, using independent sample t-test, the differences between the initial and 12-hour hs-CRP measurements (delta hs-CRP) were used to assess differences by treatment arm. A mixed model was used to detect the differential effect of treatment on the rate of change in the initial, 6-, and 12-hour hs-CRP levels, adjusting for baseline and clinical characteristics associated with initial hs-CRP measurement, and accounting for repeated measures on each participant at the different time points. We also adjusted for time from symptom onset to reperfusion therapy. The linear mixed model uses a random intercept for each subject, and for within-subject correlation. The model included terms for time, from the initiation of infusion (real-time as a continuous measurement), and an interaction term between study drug and time, in order to estimate the association between treatment arms and change in the inflammatory marker over the follow-up time points. Sensitivity analysis was done by stratifying participants based on ACS diagnosis (i.e. acute MI compared to unstable angina), and by the use of statins prior to enrollment in the study.

Univariate and multivariable linear regression models were used to study the association between hs-CRP levels at the three time points (as the predictor) and infarct size (as the outcome), after adjustment for potential confounders including age, gender, and those correlated with infarct size in univariate models at a *p*-value of  < 0.1. Infarct sizes ranged from 0 to 59 % of left ventricular mass, where 0 % represent those who did not develop an infarct, and 59 % was assigned to those who died (*n* = 4) before imaging, based on the fact that the largest infarct size measured in the study was 58 %. In addition, correlations between hs-CRP at the initial, 6-, and 12-hour determinations and infarct size were assessed using Spearman’s rank correlation. Sensitivity analyses were performed by removing participants who had no infarct to check if the association between hs-CRP and infarct size has the same direction for those who had an infarct versus those who did not. Additional sensitivity analysis was done on participants in the placebo group only to test for the correlation between hs-CRP levels, at the three time points measured, and infarct size, without the effect of GIK. We also ran sensitivity analyses based on ACS diagnosis, and prior use of statins. Because hs-CRP baseline levels were not measured prior to study infusion initiation, but rather, after hospital arrival, the time from when the drug started and time of the first hs-CRP measurement was used as a covariate in regression models.

## Results

### Characteristics of study population

A total of 143 participants met the inclusion criteria for the biocohort; 68 received GIK and 75 placebo. Not all individuals had complete hs-CRP measurements; participants with at least one hs-CRP measurement available were included in the analysis (*n* = 143). Additional file 1: Figure S1 illustrates how the biocohort participants were enrolled. Their demographic and clinical characteristics by treatment arm are shown in Table 1. The average age was 64 years in both groups; 77 % of the GIK group and 70 % of the placebo group were men. Chest pain was the chief symptom of 86 % of participants, and they were randomized at a median time of 90 min after ischemic symptom onset. The median time from start of study drug to the measurement of the initial hs-CRP values was 2.5 h in the GIK group and 2.6 h in the placebo group. The entry participant characteristics were well-balanced in the GIK and placebo groups. The clinical characteristics of participants in the biocohort were similar to all IMMEDIATE Trial participants by site of enrollment. The diagnosis of ACS was more common in the biocohort, as expected because a confirmed diagnosis of ACS was a requirement for enrollment into the biocohort (Additional file 1: Table S1).

**Table 1:** Baseline demographics and clinical characteristics of study participants by treatment group in the biocohort (*N* = 143)^a^

Cohort characteristics	No. (%)	
	*N* = 143	
	GIK (*N* = 68)	Placebo (*N* = 75)
Age, mean (SD), y	64.5 (12.9)	63.9 (12.8)
Men	52 (76.5)	52 (69.3)
Race		
White	66 (97)	71 (95)
Hispanic ethnicity	3 (4.4)	7 (9.3)
Chief complaint on presentation		
Chest pain	59 (86.8)	67 (89.3)
Shortness of breath	1 (1.5)	3 (4)
Other ^b^	8 (11.7)	5 (6.7)
Medical history		
Diabetes	12 (17.6)	19 (25.3)
Heart Failure	4 (5.9)	7 (9.3)
AMI	21 (30.9)	24 (32.0)
Medication history		
Statins	30 (44.1)	29 (38.7)
Aspirin	39 (57.3)	44 (58.7)
Minutes from symptom onset to study drug, median (IQR)	86 (51.5–160.5)	81 (53–123)
Minutes from symptom onset to study drug		
0–30	1 (1.5)	0 (0)
31–60	21 (30.9)	22 (29.3)
61–90	9 (13.2)	19 (25.3)
91–180	12 (17.7)	15 (20.0)
181–360	10 (14.7)	8 (10.7)
361–24 h	6 (8.8)	5 (6.7)
Within 24 h, unspecified	3 (4.4)	4 (5.3)
>24 h	6 (8.8)	2 (2.7)
ACI-TIPI score, mean (SD), %	83 (15.7)	83.1 (12.1)
Hospital reperfusion treatment		
Thrombolytic therapy	1 (1.5)	1 (1.3)
PCI	59 (86.8)	56 (74.7)
Coronary artery bypass graft	0 (0)	2 (2.7)
Confirmed diagnosis		
Acute myocardial infarction	58 (85.3)	68 (90.7)
Any angina	10 (14.7)	7 (9.3)
Time from study drug to biomarker measurement, median (IQR), hours		
Initial	2.5 (1.3–3.3)	2.6 (1.9–3.2)
6 h	6 (6–6.3)	6 (6–6.2)
12 h	12 (12–12.2)	12.1 (12–12.3)

*AMI* indicates acute myocardial infarction; *ACI-TIPI* acute cardiac ischemia time-insensitive predictive instrument; *GIK* glucose-insulin-potassium; *IQR* interquartile range; *PCI* percutaneous coronary intervention; *SD* standard deviation

^a^No significant differences were noted between GIK and placebo groups

^b^Abdominal pain, back pain, dizziness, heartburn, loss of consciousness, shoulder pain and weakness

### Changes in hs-CRP levels

Table 2 shows hs-CRP levels in the first 12 h of drug infusions before logarithmic transformation. Linear regression models were performed to assess the association between admission hs-CRP levels and baseline characteristics. They showed that older people, women and individuals with history of heart failure had higher hs-CRP levels upon admission (Additional file 1: Table S2). In addition the longer time from symptom onset to reperfusion therapy the higher the hs-CRP levels (Additional file 1: Table S2). These associations remained significant after adjusting for the use of study drug. No other clinical characteristics had a significant association with admission hs-CRP values.

**Table 2:** Hs-CRP Levels in the first 12 h of emergency department admission

	GIK			Placebo		
Hs-CRP	No.	Mean (SD)	Median (IQR)	No.	Mean (SD)	Median (IQR)
Initial hs-CRP mg/L	59	8.1 (17.7)	3.1 (1.4–7.3)	61	12.3 (24.4)	3.2 (1.9–9.3)
6 h hs-CRP mg/L	58	8.6 (17.0)	3.4 (1.8–8.8)	63	15.1 (30.7)	4.2 (2.0–12.4)
12 h hs-CRP mg/L	57	9.2 (16.7)	4.5 (2.3–7.9)	64	17.6 (31.0)	5.9 (3.1–13.7)

*GIK* indicates glucose-insulin-potassium; *Hs-CRP* high sensitivity C-reactive protein; *IQR* interquartile range; *SD* standard deviation

**Table 3:** Regression analysis of hs-CRP levels and 30-day infarct size^a^

	All participants			Participants with an infarct		
Regression models^b^	No.	Beta Coefficient	*P-value*	No.	Beta Coefficient	*P-value*
Initial hs-CRP mg/L^c^	83	2.3	0.46	54	3.8	0.35
6 h hs-CRP mg/L^c^	85	4.0	0.17	56	5.9	0.13
12 h hs-CRP mg/L^c^	83	6.8	0.04	56	10.6	0.02
Delta hs-CRP mg/L^d^	78	13.9	0.02	51	23.1	0.01

GIK*GIK* indicates glucose-insulin-potassium,: *Hs-CRP* high sensitivity C-reactive protein

^a^Data analyzed using logarithmically transformed hs-CRP values

^b^Adjusted for age, gender and GIK administration. In addition, the time from when the drug started to the time of the first hs-CRP measurement was used as a covariate. The coefficient represents the fitted increase in infarct size per one unit change in logarithmically trasnformed hs-CRP.

^c^Per 1 unit increase in hs-CRP levels

^d^Difference between the initial hs-CRP and 12 h hs-CRP measurements

**Fig. 1:**
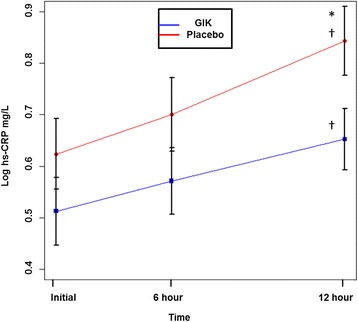
hs-CRP Levels per Treatment Arm. Time course of mean ± SEM hs-CRP at initial, 6 h and 12 h per treatment arm. *GIK* indicates glucose-insulin-potassium; *Hs-CRP* high sensitivity C-reactive protein. ^*^ *P* = 0.053 between groups at 12 h (independent sample t-test). ^†^ P˂0.01 within group differences between initial and 6 h, 6 h and 12 h, and initial and 12 h (paired sample t-test). Initial time represents the first hs-CRP measurement (median = 2.5 h)

The hs-CRP measurements increased significantly in both the control and the treatment groups by 6 and 12 h, compared with the initial measurement (*P* < 0.01 for all intragroup comparisons). Hs-CRP values were not different for those who received GIK versus placebo at the initial and 6-hour measurements; however by 12 h, the hs-CRP levels were slightly higher in placebo-treated versus GIK-treated participants (*P* = 0.053) (Fig. 1). When comparing delta hs-CRP, the differences between the initial and 12-hour hs-CRP measurements, there were no significant differences in those delta values between treatment arms (Additional file 1: Table S3). Using linear mixed model, hs-CRP levels increased significantly with time in both arms (*P* < 0.01); however, the rate of change did not differ between the GIK and placebo groups (*P* = 0.30 for time*treatment interaction). Baseline characteristics associated with initial hs-CRP were added to the model and did not change the above results. In addition, the results remained the same after adjusting for the time from symptom onset to reperfusion therapy. The results also did not change when we stratified the analysis by ACS diagnosis, and by the use of statin therapy prior to study enrollment.

### Association between hs-CRP and infarct size

After adjusting for age, gender and GIK administration (variables that were associated with infarct size in univariate models), there were no significant associations between hs-CRP and infarct size at the initial and 6-hour measurements. On the other hand the 12-hour measurement and delta hs-CRP were significantly associated with infarct size (Table 3). Sensitivity analysis done by removing participants with no infarct (i.e. those with an infarct size of 0, or those who died and did not have infarct size measurement available) (*n* = 31) showed the same association between hs-CRP levels and infarct size (Table 3). Also stratifying participants by ACS diagnosis and statin use prior to study enrollment did not significantly change the study results. Results from spearman’s rank correlation yielded similar associations between hs-CRP and 30-day infarct size (Additional file 1: Fig. S2). When testing for the correlation between hs-CRP and infarct size in the placebo group only, we found that the 12-hour hs-CRP and the delta hs-CRP remained significantly correlated with infarct size (*p*-value = 0.022 and 0.049, for the 12 h and delta hs-CRP respectively).

## Discussion

Our data confirm that plasma hs-CRP concentrations are increased in participants presenting to the ED with ACS, presumably reflecting a state of inflammation. However, the administration of GIK early in ACS did not have a significant impact on hs-CRP levels. Although there was a modest difference between the 12-hour hs-CRP levels with GIK, the delta hs-CRP values was not different between treatment arms. In addition, results of mixed models, adjusting for within and between subjects variability, demonstrated no effect of GIK on hs-CRP over the three time points. These results suggest that the beneficial effects of GIK in ACS observed in the main study [15], at least as reflected in the absence of an effect on hs-CRP, are less likely to be through an anti-inflammatory effect, and may be more extended through a metabolic effect.

Previous studies on the effect of GIK on CRP yielded conflicting results. In a study by Chaudhuri et al., GIK administration started in the emergency department in patients presenting with ST-segment elevation myocardial infarction (STEMI) (*n* = 32) and lasting 48 h showed significantly reduced hs-CRP values at 24 and 48 h post-infusion compared to placebo [18]. In contrast, Parikh et al., demonstrated in 25 patients with STEMI that a 24-hour infusion of GIK produced no statistically significant difference in 24 h hs-CRP levels compared with placebo [26]. Additionally a study by Hashemian et al., showed no effect of GIK on hs-CRP levels in 72 patients with STEMI treated within 12 h from symptom onset [27]. Although those studies added GIK to standard care, there are important differences in the use of GIK in the IMMEDIATE Trial. First, unlike prior clinical trials in which GIK was started typically an average of 6 h after onset of ischemic symptoms, following documentation of acute MI [6, 10, 14], in IMMEDIATE, the study drug was started prior to arrival to emergency department, upon emergency medical services (EMS) arrival in the community following a 9-1-1 call, at an average of 90 min after symptom onset [15]. Moreover, the previous studies only included participants with STEMI. In contrast, the IMMEDIATE Trial included participants with ACS, i.e., either unstable angina or acute MI (whether or not STEMI) [15].

Infarct size has shown to be a prognostic marker of adverse clinical outcomes after an acute coronary event [28]. Baseline CRP levels in healthy individuals or in patients with stable angina are independent risk factor for cardiovascular events [29]. Also the rise in CRP after acute MI or during unstable angina pectoris has been shown to be related to outcome [23, 30, 31]. In this study on participants presenting to EMS with ACS, we document a relationship between hs-CRP level measured at 12 h and 30-day infarct size. In addition, the magnitude of change in hs-CRP levels, between the initial and 12-hour values, was related to infarct size. Limiting the analysis to those participants with a documented infarct size measurement, and also to those in the placebo group only, showed similar results. Previous studies have shown no clear relationship between CRP levels on hospital admission and infarct size in patients with acute coronary events [32]; but nevertheless, cumulative or peak CRP levels have been correlated with infarct size [33]. Peak CRP levels are reached by no earlier than 24 h after infarction [34]. Adding to that picture, our hs-CRP measurements reflect that an early rise, within 12 h of ACS symptom onset, correlates with 30-day infarct size. Imaging studies in IMMEDIATE Trial biocohort participants at 30 days showed an 80 % reduction infarct size associated with GIK, both for the entire ACS cohort (*n* = 110) and in those presenting with STEMI (*n* = 75) [15]. Therefore the modest reduction seen in hs-CRP levels at 12 h between the two groups may be indirectly related to infarct size.

This study has several limitations. First, although our sample is larger than previous studies on the effect of GIK on hs-CRP levels [18, 26, 27]. the size of the IMMEDIATE Trial biocohort may have limited our power to detect treatment interactions. Second, CRP levels prior to the onset of GIK infusion and after the 12 h infusion were not available. Finally, although CRP is commonly used as an inflammatory biomarker, it is somewhat nonspecific and other biomarkers have been considered as an alternative to CRP. For instance, serum amyloid A, interleukin-6, and adhesion molecules such as soluble intercellular adhesion molecule type 1, similar to CRP, are markers of inflammation that are produced by the liver [35]. Therefore, if GIK exerts an anti-inflammatory effect it may be reflected through biomarkers other than CRP. Nevertheless this study has several strengths including serial hs-CRP levels measurements within 12 h after GIK initiation, compared to other studies, in which the effect of GIK on CRP was assessed at 24–48 h following treatment. Moreover, our data were collected in a randomized placebo-controlled trial, with both GIK and placebo participants having balanced characteristics.

## Conclusions

In patients with an ACS, early administration of GIK appears to have no significant effect on hs-CRP levels measured in the first 12 h of treatment infusion. This is consistent with a model that the primary immediate benefits of GIK are more likely metabolic rather than anti-inflammatory. Nevertheless, in order to more fully understand the mechanisms by which GIK exert its potential benefits in patients with an ACS, future research should consider markers of endothelial dysfunction, microvascular dysfunction, and coagulation. Our findings of a possible association between the early rise in hs-CRP levels and infarct size, are consistent with the role of inflammation in extent of infarction, an effect that deserves further investigation as a marker of acute myocardial damage and inflammation.

## Additional file

Additional file 1:
**Supplemental Material.** **Figure S1.** Participants included in the Biocohort in the IMMEDIATE Trial. **Figure S2.** Correlation between hs-CRP Levels and 30-day Infarct Size. **Table S1.** Baseline Demographic and Clinical Characteristics of Study Participants by Treatment Group Compared to the Participants from the IMMEDIATE Trial. **Table S2.** Results of Regression Analysis between Initial hs-CRP Levels and Baseline Demographic and Clinical Characteristics. **Table S3.** hs-CRP Levels per Treatment Arm. (DOCX 148 kb)

## Abbreviations

ACS: Acute coronary syndromes
CRP: C-reactive protein
EMS: Emergency medical services
FFA: Free fatty acid
GIK: Glucose-insulin-potassium
hs-CRP: High sensitivity C-reactive protein
IMMEDIATE: Immediate Myocardial Metabolic Enhancement During Initial Assessment and Treatment in Emergency care
MI: Myocardial infarction
STEMI: ST-segment elevation myocardial infarction
