# Supplementary material for: C-Reactive protein reactions to glucose-insulin-potassium infusion and relations to infarct size in patients with acute coronary syndromes
Source: BMC Cardiovasc Disord. 2015 Dec 3;15:163. doi: 10.1186/s12872-015-0153-7 (PMC4668670; doi:10.1186/s12872-015-0153-7)
Supplement: Additional file 1: — Supplemental Material. Figure S1. Participants included in the Biocohort in the IMMEDIATE Trial. Figure S2. Correlation between hs-CRP Levels and 30-day Infarct Size. Table S1. Baseline Demographic and Clinical Characteristics of Study Participants by Treatment Group Compared to the Participants from the IMMEDIATE Trial. Table S2. Results of Regression Analysis between Initial hs-CRP Levels and Baseline Demographic and Clinical Characteristics. Table S3. hs-CRP Levels per Treatment Arm. (DOCX 148 kb) [file 12872_2015_153_MOESM1_ESM.docx]

**Supplemental Material**

**Supplemental table 1.** Baseline Demographic and Clinical Characteristics of Study Participants by Treatment Group compared to the participants from the IMMEDIATE Trial^*^

| **Cohort** | **N=143** | | **N=545 ^†^** | | |  |
| --- | --- | --- | --- | --- | --- | --- |
|  | **No. (%)** | | | | |  |
| **Characteristics** | **GIK (N=68)** | **Placebo(N=75)** | | **GIK (N=256)** | **Placebo(N=272)** | |
| **Age, mean (SD), y** | 64.5 (12.9) | 63.9 (12.8) | | 64.5 (13.6) | 63.7 (13.9) | |
| **Men** | 52 (76.5) | 52 (69.3) | | 186 (72.7) | 194 (71.3) | |
| **Race** |  |  | |  |  | |
| White | 66 (97) | 71 (95) | | 214 (83.6) | 241(88.6) | |
| Black | 2 (3) | 2 (3) | | 26 (10.2) | 17 (6.3) | |
| **Hispanic ethnicity** | 3 (4.4) | 7 (9.3) | | 26 (10.2) | 34 (12.5) | |
| **Chief complaint on presentation** | | | | | | |
| Chest pain | 59 (86.8) | 67 (89.3) | | 227 (88.7) | 232 (85.3) | |
| Shortness of breath | 1 (1.5) | 3 (4) | | 6 (2.3) | 12 (4.4) | |
| Other ^‡^ | 8 (11.7) | 5 (6.7) | | 23 (9) | 28 (10.3) | |
| **Medical conditions by history** | | | | | | |
| Diabetes | 12 (17.6) | 19 (25.3) | | 79 (29.8) | 77 (28.3) | |
| Heart Failure | 4 (5.9) | 7 (9.3) | | 39 (14.7) | 44 (16.2) | |
| AMI | 21 (30.9) | 24 (32.0) | | 100 (37.7) | 96 (35.3) | |
| **Home medications** | | | | | | |
| Statins | 30 (44.1) | 29 (38.7) | | 128 (50.0) | 111(40.8) | |
| Aspirin | 39 (57.3) | 44 (58.7) | | 147 (57.4) | 148 (54.4) | |
| **Minutes from symptom onset to study drug, median (IQR)** | 86 (51.5-160.5) | 81 (53-123) | | 85.5 (50-183.75) | 82 (50-156) | |
| **Minutes from symptom onset to study drug, n (%)** | | | | | | |
| 0-30 | 1 (1.5) | 0 (0) | | 10 (4.0) | 10 (3.7) | |
| 31-60 | 21 (30.9) | 22 (29.3) | | 67 (26.7) | 72 (26.8) | |
| 61-90 | 9 (13.2) | 19 (25.3) | | 37 (14.7) | 45 (16.7) | |
| 91-180 | 12 (17.7) | 15 (20.0) | | 36 (14.3) | 40 (14.9) | |
| 181-360 | 10 (14.7) | 8 (10.7) | | 43 (17.1) | 39 (14.5) | |
| 361-24 h | 6 (8.8) | 5 (6.7) | | 19 (7.6) | 21 (7.8) | |
| Within 24 h, unspecified | 3 (4.4) | 4 (5.3) | | 18 (7.2) | 21 (7.8) | |
| >24 h | 6/68 (8.8) | 2/75 (2.7) | | 21/251 (8.4) | 21/269 (7.8) | |
| **ACI-TIPI score, mean (SD), %** | 83 (15.7) | 83.1 (12.1) | | 80.6 (18.1) | 81 (15.7) | |
| **Hospital reperfusion treatment** | | | | | | |
| Thrombolytic therapy | 1 (1.5) | 1 (1.3) | | 1 (0.4) | 3 (1.1) | |
| PCI | 59 (86.8) | 56 (74.7) | | 127 (49.6) | 131 (48.2) | |
| Coronary artery bypass graft | 0 (0) | 2 (2.7) | | 6 (2.3) | 10 (3.7) | |
| **Confirmed diagnosis** | | | | | | |
| AMI | 58 (85.3) | 68 (90.7) | | 139 (54.3) | 161 (59.2) | |
| Any angina | 10 (14.7) | 7 (9.3) | | 33 (12.9) | 32 (11.8) | |
| Non-Ischemic cardiac disease | 0 (0) | 0 (0) | | 36 (14.1) | 29 (10.7) | |
| Non-cardiac disease | 0 (0) | 0 (0) | | 48 (18.8) | 50 (18.4) | |

AMI, acute myocardial infarction; ACI-TIPI, acute cardiac ischemia time-insensitive predictive instrument; GIK indicates glucose-insulin-potassium; IQR, interquartile range; PCI, percutaneous coronary intervention; and SD, standard deviation.

^*^ No significant differences were noted between biocohort and participants from the IMMEDIATE Trial.

^†^ Participants enrolled in same centers as the biocohort.

^‡^ Abdominal pain, back pain, dizziness, heartburn, loss of consciousness, shoulder/arm pain and weakness.

**Supplemental table 2.** Results of Regression Analysis between Initial hs-CRP Levels and Baseline Demographic and Clinical Characteristics^*^

| **Baseline Characteristics** | **Beta Coefficient** | ***P-value*** |
| --- | --- | --- |
| **Age** | 0.01 | 0.05 |
| **Gender** | -0.27 | 0.02 |
| **History of Diabetes Mellitus** | 0.16 | 0.22 |
| **History of Heart Failure** | 0.57 | 0.01 |
| **History of Myocardial Infarction** | 0.11 | 0.32 |
| **Minutes from Symptom Onset to Reperfusion Therapy** | 0.0003 | 0.04 |

Hs-CRP indicates high sensitivity C-reactive protein.

^*^ Data analyzed using logarithmically transformed hs-CRP values

**Supplemental table 3.** hs-CRP Levels per Treatment Arm^*^

| **hs-CRP mg/L** | **GIK** | **Placebo** | **P-value** |
| --- | --- | --- | --- |
| Initial, mean (SD) | 0.51 (0.54) [n=59] | 0.62 (0.60) [n=61] | 0.29 |
| 6 hour, mean (SD) | 0.57 (0.53) [n=58] | 0.70 (0.62) [n=63] | 0.22 |
| 12 hour, mean (SD) | 0.65 (0.50) [n=57] | 0.84 (0.58) [n=64] | 0.053 |
| Delta^†^, mean (SD) | 0.15 (0.25) [n=56] | 0.20 (0.38) [n=57] | 0.41 |

GIK indicates glucose-insulin-potassium; and Hs-CRP indicates high sensitivity C-reactive protein.

^*^ Data analyzed using logarithmically transformed hs-CRP values

^†^ Difference between the initial hs-CRP and 12 hour hs-CRP measurements.

**Supplemental figure 1.** Participants included in the Biocohort the IMMEDIATE Trial

871 Enrolled to receive GIK or placebo in the IMMEDIATE Randomized Controlled Trial

411 Randomized to receive GIK

460 Randomized to receive placebo

104 Excluded (ED diagnosis non-ACS, study drug discontinued)

102 Excluded (ED diagnosis non-ACS, study drug discontinued)

307 Included in modified ITT group

358 Included in modified ITT group

7 Declined consent to biocohort

199 Were not asked to participate in biocohort (non-enrolling period)

33 Not included (received <8 hours of study drug and/or did not have ACS)

8 Declined consent to biocohort

248 Were not asked to participate in biocohort (non-enrolling period)

26 Not included (received <8 hours of study drug and/or did not have ACS)

ACS indicates acute coronary syndrome; ED, emergency department; GIK; glucose-insulin-potassium; and ITT, intent to treat analysis.

**Supplemental figure 2.** Correlation between hs-CRP Levels and 30-day Infarct Size*


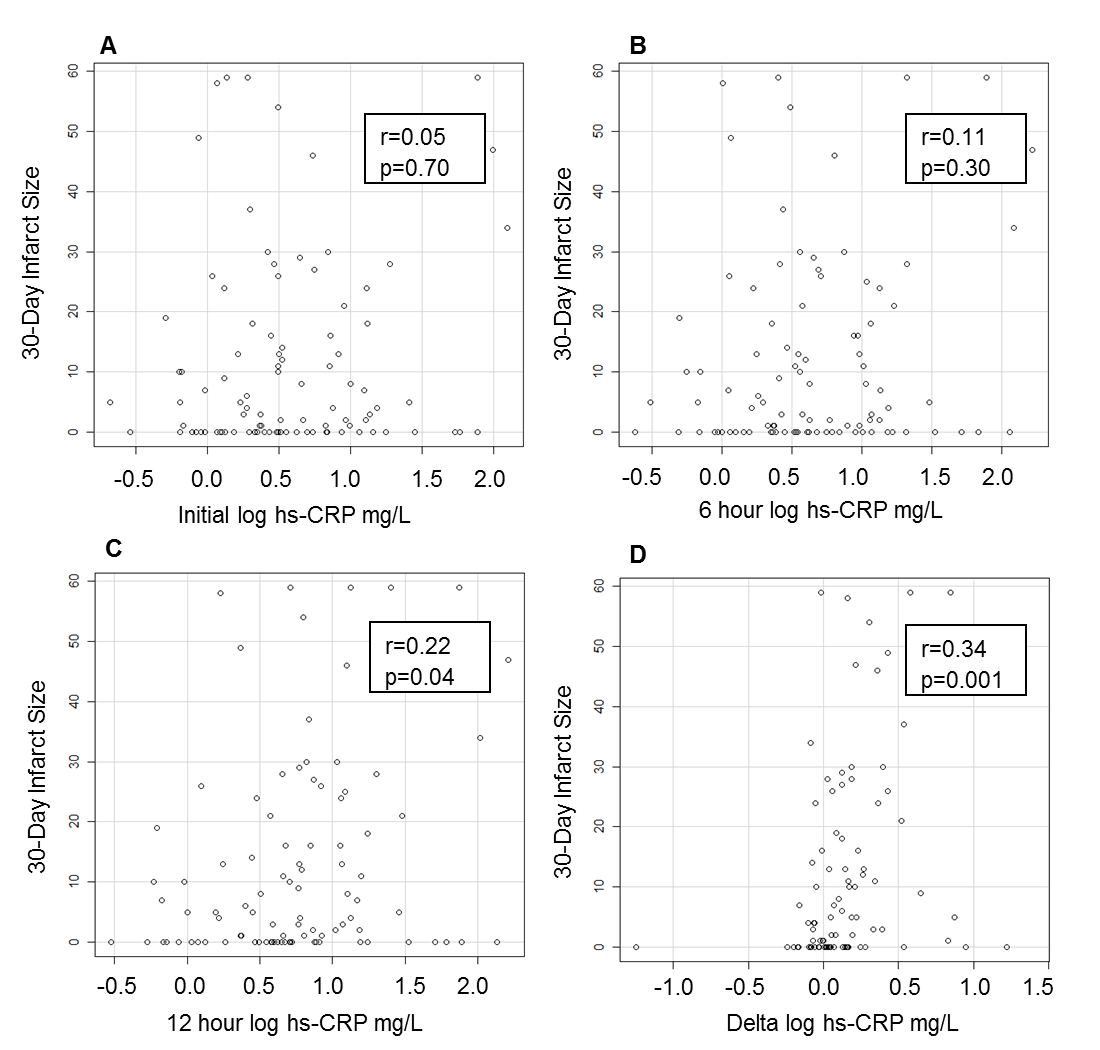


Correlation was evaluated by Spearman's rank correlation method. A. Correlation between 30-day infarct size and initial hs-CRP. B. Correlation between 30-day infarct size and six hours hs-CRP. C. Correlation between 30-day infarct size and 12 hours hs-CRP. D. Correlation between 30-day infarct size and delta hs-CRP.

Hs-CRP indicates high sensitivity C-reactive protein.

^*^ Data analyzed using logarithmically transformed hs-CRP values
